# *Leishmania* infection induces a limited differential gene expression in the sand fly midgut

**DOI:** 10.1186/s12864-020-07025-8

**Published:** 2020-09-04

**Authors:** Iliano V. Coutinho-Abreu, Tiago Donatelli Serafim, Claudio Meneses, Shaden Kamhawi, Fabiano Oliveira, Jesus G. Valenzuela

**Affiliations:** Vector Molecular Biology Section, Laboratory of Malaria and Vector Research, National Institute of Allergy and Infectious Diseases, National Institutes of Health, Rockville, MD USA

**Keywords:** Sand fly, Midgut, RNA-Seq, Transcriptomics, *Lutzomyia longipalpis*, *Leishmania infantum*, Transcriptome, Vector, Parasite

## Abstract

**Background:**

Sand flies are the vectors of *Leishmania* parasites. To develop in the sand fly midgut, *Leishmania* multiplies and undergoes various stage differentiations giving rise to the infective form, the metacyclic promastigotes. To determine the changes in sand fly midgut gene expression caused by the presence of *Leishmania*, we performed RNA-Seq of uninfected and *Leishmania infantum*-infected *Lutzomyia longipalpis* midguts from seven different libraries corresponding to time points which cover the various *Leishmania* developmental stages.

**Results:**

The combined transcriptomes resulted in the de novo assembly of 13,841 sand fly midgut transcripts. Importantly, only 113 sand fly transcripts, about 1%, were differentially expressed in the presence of *Leishmania* parasites. Further, we observed distinct differentially expressed sand fly midgut transcripts corresponding to the presence of each of the various *Leishmania* stages suggesting that each parasite stage influences midgut gene expression in a specific manner. Two main patterns of sand fly gene expression modulation were noted. At early time points (days 1–4), more transcripts were down-regulated by *Leishmania* infection at large fold changes (> 32 fold). Among the down-regulated genes, the transcription factor Forkhead/HNF-3 and hormone degradation enzymes were differentially regulated on day 2 and appear to be the upstream regulators of nutrient transport, digestive enzymes, and peritrophic matrix proteins. Conversely, at later time points (days 6 onwards), most of the differentially expressed transcripts were up-regulated by *Leishmania* infection with small fold changes (< 32 fold). The molecular functions of these genes have been associated with the metabolism of lipids and detoxification of xenobiotics.

**Conclusion:**

Overall, our data suggest that the presence of *Leishmania* produces a limited change in the midgut transcript expression profile in sand flies. Further, *Leishmania* modulates sand fly gene expression early on in the developmental cycle in order to overcome the barriers imposed by the midgut, yet it behaves like a commensal at later time points where a massive number of parasites in the anterior midgut results only in modest changes in midgut gene expression.

## Background

*Leishmania* is a digenetic parasite developing in a mammalian host as well as in an insect vector. These parasites are mostly transmitted by phlebotomine sand flies (Diptera: Psychodidae) of the genera *Phlebotomus* and *Lutzomyia* in the Old and New Worlds, respectively [1].

*Leishmania* develops in the lumen of the sand fly midgut [2–4]. Once a sand fly takes up an infected blood meal, round-shaped *Leishmania* amastigotes (mammalian stage) are carried along within macrophages into the midgut lumen. Between 18 h and 24 h post blood meal, these parasites are released from the macrophages and start to differentiate into procyclic promastigotes within blood enveloped by the peritrophic matrix [5]. During this process, the parasites elongate their cell bodies and expose their flagella, becoming fully differentiated into procyclics by day 2. Between days 2 and 4, *Leishmania* multiplies and undergoes another differentiation step, acquiring an elongated form (banana-like shape) termed nectomonads [2–4]. Upon the breakdown of the peritrophic matrix, the nectomonads escape to the ectoperitrophic space and eventually dock on the midgut microvilli [6, 7]. As the remains of the digested blood are evacuated, the parasites detach from the epithelium and further differentiate into the leptomonad stage, which exhibit a smaller cell body and a longer flagellum than nectomonads [2–4]. From day 6 onwards, the leptomonads undergo a differentiation process, termed metacyclogenesis, giving rise to the infective forms, the metacyclic promastigotes [8]. During metacyclogenesis, the parasites replace their glycocalyx, exhibiting different sugar side chains on their major surface glycans, reduce the size of their cell bodies, and elongate their flagella [2–4]. All these transformations give rise to free swimming highly motile parasites [2–4].

Even when developing in their natural sand fly vectors, *Leishmania* faces barriers imposed by the midgut; overtaking such barriers is critical for the development of mature *Leishmania* infections. During the transitional stages between amastigotes and procyclic promastigotes, the parasites are susceptible to the harmful action of digestive enzymes [9]. The sand fly immune system may also counteract infection with the parasites, by activation of the Imd pathway [10, 11]. Escaping from the peritrophic matrix (PM) is also a crucial step for *Leishmania* survival [12, 13]. Another critical barrier to development is attachment of *Leishmania* parasites to the midgut epithelium [14]. For this step, specific carbohydrate side chains are required for binding to a midgut epithelium receptor [7, 15, 16]. From there on, undefined parameters trigger the metacyclogenesis process in parasites leading to the development of a mature infection.

The midgut transcriptomes of three sand fly species have been described. These focused mostly on differences in gene expression triggered by blood intake and parasite infection as compared to sugar fed midguts [17–19]. Nonetheless, such studies took place before the advent of deep sequencing and were limited to the investigation of about 1000 transcripts due to the low dynamic range of cDNA libraries. Despite such a limited pool of genes, these studies unveiled multiple genes differentially regulated by blood and/or *Leishmania* infection. For the later, genes encoding digestive enzymes and components of the peritrophic matrix, the main midgut barriers to *Leishmania* development, were differentially regulated [17–19]. A broader transcriptome of *Phlebotomus papatasi* cDNA libraries, encompassing multiple stages and time points post-blood feeding, identified 17,120 transcripts and annotated transcripts encoding proteins participating in digestion, PM processes, and immunity [20].

In order to investigate the effects of *Leishmania* infection on sand fly midgut gene expression, we carried out an RNA-Seq analysis of *Leishmania infantum*-infected *Lutzomyia longipalpis* midguts at 7 time points, each corresponding to when the insect midguts are enriched with a particular *Leishmania* developmental stage. These encompassed early time points when blood digestion is taking place as well as late time points when the parasites are undergoing metacyclogenesis. This approach expands our breadth of knowledge by assessing the effect of *Leishmania* infection on over 13,000 sand fly midgut transcripts, focusing on genes encoding secreted proteins and also on genes participating in other biological processes of midgut epithelial cells.

## Results

### Sand fly infection and *Leishmania* differentiation

*Le. infantum* growth in the *Lu. longipalpis* sand fly midgut followed a typical and expected pattern [21]. Briefly, a median of 3000 parasites detected early at 4d Pi increased to 6000 parasites by day 6 and reached about 126,000 parasites at 14d. The proportion of metacyclic stage parasites increased from 0% on 6d to 92% on 14d.

### Expanding the *Lu. longipalpis* midgut repertoire of putative proteins

We obtained a total of 53,683,499 high quality *Lu. longipalpis* midgut-specific reads from the de novo assembly of seven libraries prepared from RNA extracted from uninfected midguts at 1d, 2d, 4d, 6d, 8d, 12d and 14d. High quality reads were assembled in 57,016 contigs that were further filtered to 13,841 putative contigs based on the presence of an open reading frame (ORF) and on similarities to proteins deposited at Refseq invertebrate, NCBI Genbank or SwissProt databases (Additional file 1: Table S1). We also searched for putative secreted proteins where a signal peptide was predicted. The contigs or transcripts from these libraries had a mean size of 1498 bp, with the shortest comprising 150 bp and the longest 27,627 bp. Overall, 72% were categorized to a functional class after BLAST analysis (e < 10E-6) against nine distinct databases (Additional file 2: Figure S1 and Additional file 3: Table S2). Unknown contigs accounted for 28% of contigs, but only for 6.56% of transcriptome abundance.

**Fig. 1:**
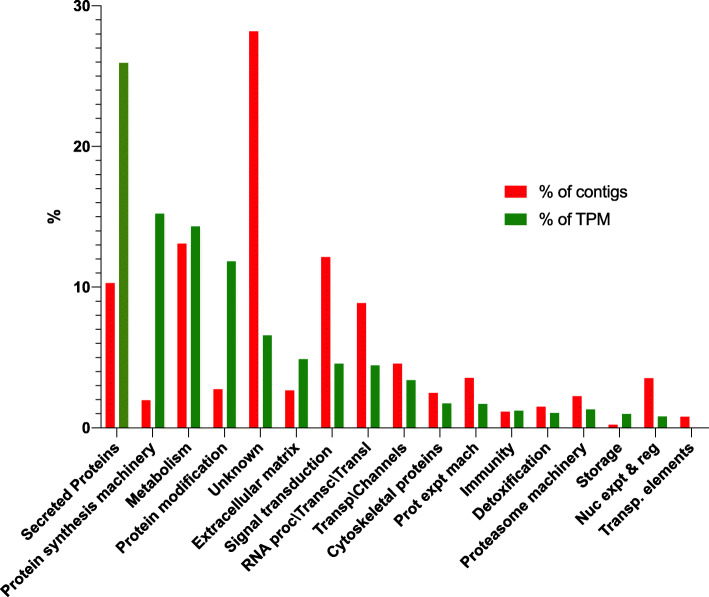
Overview of the transcriptome repertoire displaying the overall percentage of contigs (% of contigs) and abundance (%TPM) for all time points. The distribution of the mapped reads to the functional classification are highlighted

The annotations of all the 13,841 sand fly midgut transcripts from this work are described in Table S1 (Additional file 1: Table S1), where the best match of each sequence to NCBI, KOG, and Swiss protein databases are shown in different columns. Table S1 also describes if the protein is potentially secreted by giving the term “SIG” in the SigP Result column as a result of SignalP analysis of all transcripts. Using this information, we classified the transcripts into functional categories. The most represented functional categories were secreted proteins with 25.9% of transcripts per million (TPM), protein synthesis (15.2% of TPM), metabolism (14.3% of TPM) and protein modification (11.8% of TPM; Fig. 1 and Additional file 3: Table S2). Importantly, only 0.59% of all transcripts did not have a match in any of the databases tested, indicating that more than 99% of the transcripts from this transcriptome are insect specific transcripts (Additional file 1: Table S1).

The search for transcripts encoding protein families potentially participating in biological processes important for midgut physiology and *Leishmania* development resulted in 740 sequences encoding proteins associated with immune responses, digestion, and chitin metabolism (Additional file 4: Table S3).

Among the immune-related genes (194 transcripts), the major components of the Toll-like receptor and Imd pathways participating in recognition (GNBPs and PGRPs), signal transduction (Spatzle and TAK1), regulation (cactus and caspar), transcription factors (dorsal and relish), as well as effector molecules (antimicrobial peptides) were identified (Additional file 4: Table S3). Members of the Reactive Oxygen Species (ROX)-producing MAP kinase pathway, such as DUOX, were also identified (Additional file 4: Table S3). Multiple transcripts encoding proteins associated with ROX metabolism (oxidative stress), cell death (JNK pathway and apoptosis) epithelium regeneration (JAK-STAT pathways) were also pinpointed (Additional file 4: Table S3).

Regarding the digestive enzymes (348 transcripts), 142 serine protease-encoding transcripts were identified, of which 33 and 22 transcripts encoded trypsins and chymotrypsins, respectively (Additional file 4: Table S3). Amongst the 55 carbohydrases, 24 amylases and 6 glucosydases were identified (Additional file 4: Table S3). Furthermore, 49 carboxypeptidases, 41 aminopeptidases, 56 lipases, and 5 nucleotidases were identified (Additional file 4: Table S3).

The 198 transcripts related to chitin metabolism are possibly involved with the synthesis, scaffolding, modification, and degradation of the PM (Additional file 4: Table S3). Regarding chitin synthesis, two transcripts encoding chitin synthase were identified (Additional file 4: Table S3). Transcripts encoding PM-scaffolding proteins encompassed 28 peritrophins and 7 of 25 chitin-binding protein of the CPAP subgroup (Cuticular proteins analogous to peritrophins), which displayed relevant expression levels in the midgut (Additional file 4: Table S3). Regarding PM modification, 4 transcripts encoding chitin deacetylases were pinpointed (Additional file 4: Table S3). For PM degradation and chitin digestion, 15 and 6 transcripts encoding chitinases and N-acetyl-glucosaminidases, respectively, were identified (Additional file 4: Table S3).

Compared to the annotation of the *P. papatasi* cDNA libraries of whole bodies and multiple stages and time points post-blood feeding [20], multiple additional homolog transcripts were identified in the *Lu. longipalpis* midgut RNA-Seq libraries, including transcripts encoding PGRPs, C-type lectins, and lysozymes amongst the immune genes. Also, the RNA-Seq libraries displayed more matches of sequences encoding all classes of digestive enzymes (except aminopeptidases), multiple peritrophins, and twice as many chitinase sequences (Table 1).

**Table 1:** Comparison of number of transcript matches amongst gene families between *Phlebotomus papatasi* [20] and *Lutzomyia longipalpis* (current study)

Gene families	*P. papatasi* cDNA [20]	*L. longipalpis* RNA-Seq (Current study)
	**Immunity**	
**PGRP**	2	7
**PGRP-SC**	3	1
**BGRP**	3	4
**CTL**	7	15
**Scavenger receptor**	9	10
**Galectins**	7	4
**TEP**	3	2
**Spätzle**	5	4
**Pellino**	1	1
**IAP2**	5	1
**Fos**	3	3
**Jun**	2	1
**prophenoloxidases**	4(10)^b^	4
**lysozymes**	2	10
**hemomucin**	3	2
	**Digestive Enzymes**	
**trypsins**	9	33
**Chymotrypsins**	20	22
**Aminopeptidases**	45	41
**Carboxypeptidases**	18	49
**Glucosidases**	23	6(31)^a^
**Amylases**	8	24
**Lipases**	31	56
	**PM/Chitin**	
**Peritrophins**	1(4)^b^	28
**Chitin synthase**	4(9)^b^	2
**Chitinase**	8	15

^a^(non-amylase matches)

^b^(sequences encompassing alleles of the same gene)

### Sand fly midgut gene expression

The obtained midgut transcriptome dataset was used to determine the sand fly midgut differential expression caused by *Leishmania* infection. All transcripts used for this analysis can be found in Table S1 (Additional file 1: Table S1.).

**Fig. 2:**
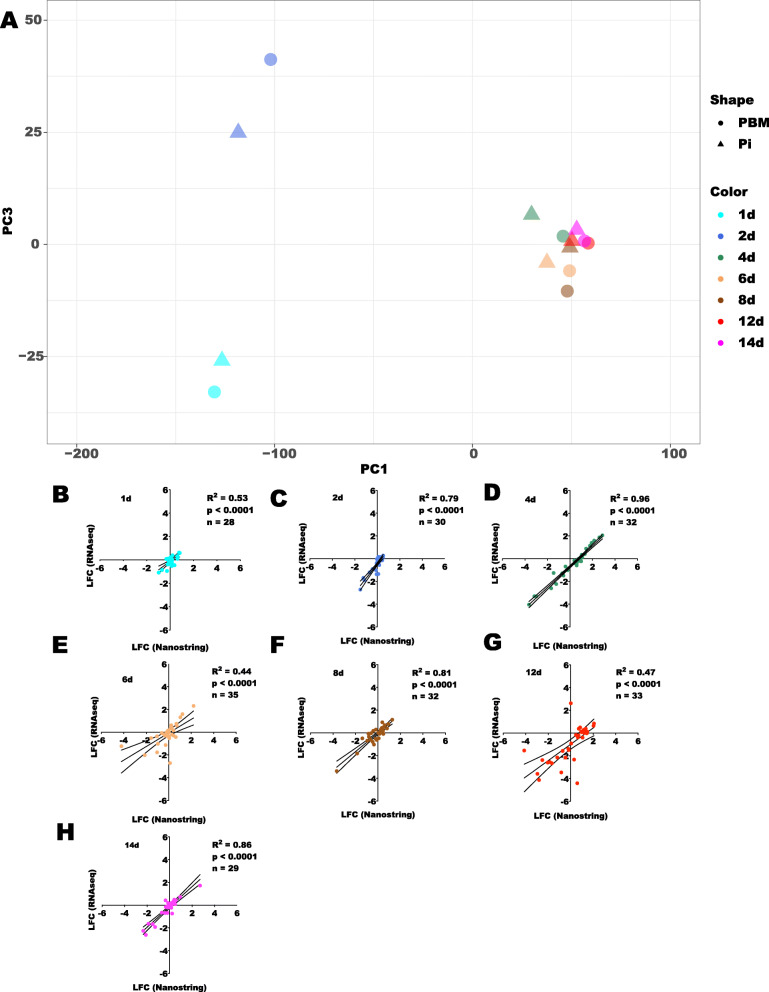
Midgut sequencing overall analysis. **a**. Principal component analysis (PCA) describing the position of each midgut time point on the expression space. Expression space was generated based on the log_2_ of TPMs using the 10,000 most highly expressed transcripts across libraries. The Eigenvalues and % variance for PC1 and PC3 were 6221.99 and 77.19% and 330.34 and 4.1%, respectively. **b-h.** Gene expression validation by nCounter (Nanostring). Linear regression analyses comparing the expression profiles of randomly chosen transcripts obtained with RNA-Seq and nCounter (Nanostring) techniques for the seven time points. All comparisons were statistically significant (*p* < 0.0001). R^2^: regression coefficient. n: number of transcripts. The color codes labeling each time point were as follow: Aqua (1d); Royal Blue (2d); Sea Green (4d); Sandy Brown (6d); Saddle Brown (8d); Red (12d); and Fuchsia (14d). The triangle and circle shapes represent *Leishmania*-infected and uninfected samples, respectively

We performed a Principal Component Analysis (PCA) to summarize the overall expression profiles of the infected and uninfected midgut transcripts from the seven time points that represent infected midguts enriched with a different *Leishmania* stage (Fig. 2a and Additional file 5: Table S4) as well as amongst replicates (Additional file 6: Figure S2A-B and Additional file 5: Table S4). The PC1 axis shows a clear separation of transcripts between the midguts in which blood digestion was ongoing (Fig. 2a left side, 1d PBM/Pi and 2d PBM/Pi) and less separation from the time points at which the blood was mostly digested (Fig. 2a right side, 4d PBM/Pi) and from the remaining time points where the midguts were clear of blood (Fig. 2a right side, 6d to 14 PBM/Pi). The PC1 accounted for 77.2% of the variance (Additional file 5: Table S4). On the other hand, the PC3, rather than PC2 (Additional file 6: Figure S2A-B), depicted the separation between infected from the uninfected samples (Fig. 2a), accounting for 4.1% of the variance (Additional file 5: Table S4).

The expression profiles of midgut transcripts were validated by assessing the expression levels of selected midgut genes (*n* = 28–35; Additional file 7: Table S5) using the nCounter technology (NanoString). The mean log_2_ fold change (LFC) of infected over uninfected samples was compared at each time point with LFC data obtained by RNA-Seq for the same genes. Representative genes participating in chitin metabolism/peritrophic matrix scaffolding (peritrophins and chitinases), immunity (defensin, catalase, and spatzle), digestion (amylase and chymotrypsin) among others are depicted in Fig. 2b-h. The regression analyses between the expression levels obtained with nCounter and RNA-Seq were statistically significant (*p* < 0.0001) for all seven time points (Fig. 2b-h), and the regression coefficients were greater than 0.5 for all time points, except 6d (R^2^ = 0.40) and 12d (R^2^ = 0.47) as shown in Fig. 2b-h.

### Minimal modulation of sand fly midgut gene expression by *Leishmania* infection

**Fig. 3:**
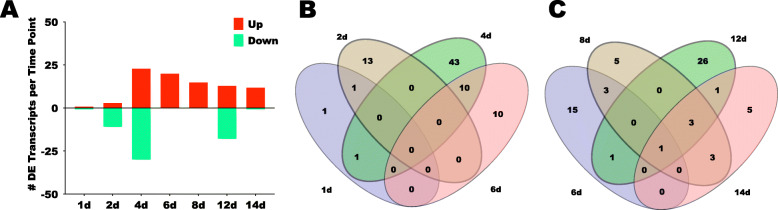
Analysis of differentially expressed (DE) midgut transcripts across time points. **a**. Number of DE transcripts up- and down-regulated in *Leishmania* infected over uninfected midguts at each time point. **b**. Venn diagrams depicting the number of DE transcripts unique and shared amongst the time points 1d through 6d. **c**. Venn diagrams depicting the number of DE transcripts unique and shared amongst time points 6d through 14d

Differences in midgut gene expression between *Leishmania*-infected over uninfected midguts were assessed. Overall, such differences accounted for only 113 differentially expressed transcripts (1 < LFC > 1; q-value < 0.05). The number of DE transcripts gradually increased from 2 transcripts on 1d to 53 transcripts on 4d (Fig. 3a). Thereafter, the number of DE transcripts decreased to 20 transcripts on 6d, and 15 transcripts on 8d (Fig. 3a). 4 days later, there was a strong increase in the number of DE transcripts (12d = 32 transcripts), which was reduced to 13 transcripts 2 days later at 14d (Fig. 3a).

Amongst the midgut genes differentially expressed upon *Leishmania* infection, some appear to play a role in specific biological processes (Table 2 and Additional file 8: Table S6). A gene encoding the transcription factor Forkhead/HNF-3 (lulogut44569) was down-regulated on 2d. Genes encoding proteins potentially involved with metabolism of steroid hormones, such as 17-beta-hydroxysteroid dehydrogenase 13-like (lulogut32574) and juvenile hormone esterase (lulogut40195) were down-regulated on 2d; a putative juvenile hormone binding protein (lulogutSigP-24,104) was down-regulated on 4d; and an ecdysteroid kinase (lulogut41307) was down-regulated on 12d. Also, genes encoding a peritrophic matrix protein (lulogutSigP-40,401), involved with the peritrophic matrix scaffolding, the antimicrobial peptide attacin (lulogutSigP-8812), and amino acid (lulogut16004) and trehalose (lulogutSigP-40,100) transporters, were down-regulated on 4d. Amongst the up-regulated genes, multiple peptidases and proteases were up-regulated on 4d and 6d. Likewise, multiple insect allergen proteins (microvilli proteins) of unknown function were up-regulated on 4d and 6d upon *Leishmania* infection. From 8d onwards, multiple cytochrome p450 transcripts were upregulated.

**Table 2:** Selected midgut transcripts differentially regulated upon *Leishmania* infection

Transcript name	Putative Encoded Protein	E-value	Time-Point(s)	Up/Down Regulated
**lulogut44569**	Forkhead/HNF-3-related transcription factor	0	2d	Down
**lulogut32574**	17-beta-hydroxysteroid dehydrogenase 13-like isoform X2	8E-66	2d	Down
**lulogut40195**	juvenile hormone esterase	6.00E-29	2d	Down
**lulogutSigP-24,104**	JAV08889.1 juvenile hormone binding protein	0	4d	Down
**lulogutSigP-40,401**	Chitin binding Peritrophin-A	4.00E-12	4d	Down
**lulogutSigP-8812**	attacin precursor	5.00E-64	4d	Down
**lulogut16004**	Amino acid transporters	0	4d	Down
**lulogutSigP-40,100**	Facilitated trehalose transporter Tret1	5.00E-93	4d	Down
**lulogutSigP-25,516**	chymotrypsin-2	8.00E-80	4d	Up
**lulogutSigP-33,169**	Trypsin-like serine protease	4.00E-67	4d	Up
**lulogutSigP-12,857**	carboxypeptidase A	0	4d/6d	Up
**lulogutSigP-53,922**	Secreted metalloprotease	0	6d	Up
**lulogutSigP-646**	Insect allergen related repeat	5.00E-28	4d	Up
**lulogutSigP-16,736**	Insect allergen related repeat	4.00E-30	4d/6d	Up
**lulogutSigP-13,949**	Insect allergen related repeat	2.00E-42	4d/6d	Up
**lulogutSigP-13,652**	Insect allergen related repeat	2.00E-32	4d/6d	Up
**lulogutSigP-54,492**	Insect allergen related repeat	5.00E-42	6d	Up
**lulogutSigP-8474**	probable cytochrome P450 6a14	0	8d/12d/14d	Up
**lulogut46050**	cytochrome P450 4C1	0	8d	Up
**lulogut33084**	Cytochrome P450 CYP3/CYP5/CYP6/CYP9 subfamilies	0	12d	Up
**lulogut34615**	probable cytochrome P450 6d5	0	8d/14d	Up
**lulogut41307**	JAV11511.1 ecdysteroid kinase	0	12d	Down

The presence of *Leishmania* in the midgut led to more genes being down-regulated at d2 and up-regulated at later time points, except on 12d (Fig. 3a and Additional file 8: Table S6). On 1d, 2d, and 4d, the early time points, 1, 11, and 30 genes were down-regulated (Fig. 3a and Table 3 and Additional file 8: Table S6) whereas 1, 3, and 23 genes were up-regulated (Fig. 3a and Table 3 and Additional file 8: Table S6). On 6d and 8d, on the other hand, 20 and 15 genes were up-regulated, yet none were down-regulated (Fig. 3a). Infected midguts on day 12 displayed 13 up-regulated compared to 18 down-regulated genes (Fig. 3a). The 14d time point exhibited more up-regulated (12 genes) than down-regulated (1 gene) genes in infected over uninfected midguts (Fig. 3a).

**Table 3:** Top eight up-regulated midgut transcripts upon *Leishmania* infection per time point

Time Point	Class	Transcript name	Putative Encoded Protein	E-value	LFC
**1d**	tr	lulogutSigP-46,620	Permease of the major facilitator superfamily	9.00E-85	6.036
**2d**	pe	lulogut42669	Endosomal membrane EMP70–10 predicted membrane helices	0	6.449
	met/aa	lulogut42063	Glutamate decarboxylase	0	2.361
	prot	lulogut44776	E3 ubiquitin-protein ligase listerin-like	0	1.508
**4d**	imm	lulogutSigP-25,698	Major epididymal secretory protein HE1 - signalP detected	3.00E-12	2.443
	s/	lulogutSigP-646	Insect allergen related repeat - signalP detected	5.00E-28	2.306
	s/	lulogutSigP-16,736	Insect allergen related repeat - signalP detected	4.00E-30	2.223
	s/	lulogutSigP-13,949	Insect allergen related repeat - signalP detected	2.00E-42	2.164
	s/	lulogutSigP-32,546	Secreted metalloprotease	0	2.021
	met/carb	lulogut24944	Alpha-L-fucosidase - signalP detected	0	1.843
	s/	lulogutSigP-13,652	Insect allergen related repeat - signalP detected	2.00E-32	1.779
	met/aa	lulogutSigP-33,280	Puromycin-sensitive aminopeptidase - signalP detected	0	1.761
**6d**	s/	lulogutSigP-54,492	Insect allergen related repeat - signalP detected	5.00E-42	2.445
	s/	lulogutSigP-53,922	Secreted metalloprotease	0	2.404
	s/	lulogutSigP-32,546	Secreted metalloprotease	7.00E-29	2.312
	pm	lulogutSigP-35,736	Trypsin-like serine protease - signalP detected	0	2.177
	pm	lulogut24040	Peptide methionine sulfoxide reductase	2.00E-58	2.102
	pm	lulogutSigP-1870	Trypsin-like serine protease - signalP detected	5.00E-67	1.842
	detox	lulogut45589	JAV13729.1 glutathione s-transferase	0	1.836
	s/	lulogutSigP-13,652	Insect allergen related repeat - signalP detected	2.00E-32	1.759
**8d**	pm	lulogutSigP-35,736	Trypsin-like serine protease - signalP detected	2.00E-58	1.719
	met/aa	lulogutSigP-39,956	Puromycin-sensitive aminopeptidase - signalP detected	0	1.642
	detox/ox	lulogut46050	XP_001843663.1 cytochrome P450 4C1	0	1.484
	detox/ox	lulogut36308	probable cytochrome P450 6a14	0	1.368
	met/lipd	lulogut34584	XP_001651935.1 epoxide hydrolase 1	5.00E-92	1.363
	detox	lulogut45588	JAV13724.1 glutathione s-transferase-like protein	3.00E-77	1.353
	detox/ox	lulogutSigP-48,117	probable cytochrome P450 6a14	0	1.173
	detox/ox	lulogut15028	XP_001870174.1 cytochrome P450 6a8	0	1.145
**12d**	detox/ox	lulogut32543	XP_001870174.1 cytochrome P450 6a8	0	1.592
	met/nuc	lulogut42037	JAV11176.1 alkaline nuclease partial	0	1.307
	met/lipd	lulogut50375	Long chain fatty acid acyl-CoA ligase	4.00E-52	1.252
	detox	lulogut33084	Cytochrome P450 CYP3/CYP5/CYP6/CYP9 subfamilies	0	1.221
	pe	lulogutSigP-54,446	Peptide exporter ABC superfamily	3.00E-59	1.189
	detox/ox	lulogutSigP-8474	probable cytochrome P450 6a14	0	1.171
	detox	lulogutSigP-34,911	Cytochrome P450 CYP3/CYP5/CYP6/CYP9 subfamilies	1.00E-59	1.107
	detox/ox	lulogut237	XP_001649312.1 probable cytochrome P450 6d5	1.00E-68	1.093
**14d**	met/carb	lulogut56076	JAV12467.1 udp-glucoronosyl and udp-glucosyl transferase	0	2.140
	detox	lulogut13235	ABV44726.1 glutathione S-transferase-like protein	2.00E-88	1.692
	detox/ox	lulogutSigP-8474	probable cytochrome P450 6a14	0	1.359
	met/lipd	lulogut34584	XP_001651935.1 epoxide hydrolase 1	5.00E-92	1.258
	detox/ox	lulogut32543	XP_001870174.1 cytochrome P450 6a8	0	1.239
	met/lipd	lulogutSigP-34,488	Acyl-CoA synthetase - probable fragment - signalP detected	7.00E-83	1.217
	met/carb	lulogutSigP-34,624	JAV12537.1 udp-glucoronosyl and udp-glucosyl transferase	0	1.181
	detox/ox	lulogut237	XP_001649312.1 probable cytochrome P450 6d5	1.00E-68	1.174

Legends: *Detox* oxidative metabolism/detoxification, *Imm* immunity, *Met* metabolism, *Pe* protein export, *Pm* protein modification, *Prot* proteosome machinery, *Tr* transporters and channels, *Glutat* glutathione s-transferase, *Oxidase* oxidase/peroxidase, *Aa* amino acid metabolism, *Carb* carbohydrate metabolism, *Lipd* lipid metabolism, *Nuc* nucleotide metabolism. *S/* other, *Uk* unknown protein. *LFC* log_2_ Fold Change

Venn diagrams show the number of DE genes at unique time points, as compared to the number of DE genes shared by multiple time points (Fig. 3b-c and Additional file 9: Table S7). In the comparisons between early time points (1d through 6d; Fig. 3b), 1 out of the 2 DE genes on 1d was only modulated at that time point (Fig. 3b). Similarly, 13 out of the 14 genes, and 43 out of 54 genes, were uniquely DE on 2d and 4d, respectively (Fig. 3b). Only the 6d DE genes exhibited as many unique as shared with 4d DE genes (10 genes; Fig. 3b). The comparisons of DE genes between later time points (6d through 14d) showed a greater number of shared DE genes between time points (Fig. 3c). For instance, only 5 out of 15, and 5 out of 13, DE genes were unique to 8d and 14d, respectively (Fig. 3c). The 12d midguts, on the other hand, exhibited 26 uniquely expressed genes out 32, the most amongst the late time points (Fig. 3c).

### Patterns of differentially expressed genes across time points

Most of the midgut genes DE by *Leishmania* infection were up-regulated by up to 32-fold (LFC < 5; Table 3; Additional file 10: Figure S3; Additional file 11: Table S8). These DE genes encoded multiple digestive enzymes and allergen-related peptides at early time points and detoxification-related proteins at later time points (Table 3; Additional file 10: Figure S3). On the other hand, multiple genes were down-regulated in *Leishmania*-infected midguts by more than 32-fold (LFC > − 5; Table 4; Additional file 10: Figure S3; Additional file 11: Table S8). Regarding the midgut genes downregulated by *Leishmania* infection (Table 4 and Additional file 10: Figure S3), none were DE on 6d and 8d (Table 4). Such genes encode a variety of proteins of unknown function as well as proteins involved in the lipid metabolism (Table 4).

**Table 4:** Top five down- regulated midgut transcripts upon *Leishmania* infection per time point

Time Point	Class	Transcript name	Putative Encoded Protein	E-value	LFC
**1d**	nr	lulogut42801	DNA damage-responsive repressor GIS1/RPH1 jumonji superfamily	0	−1.419
**2d**	st	lulogut40195	NP_523758.3 juvenile hormone esterase isoform A	6.00E-29	−1.823
	tr	lulogutSigP-32,510	Permease of the major facilitator superfamily	0	−1.9194
	tr	lulogutSigP-46,620	Permease of the major facilitator superfamily	9.00E-85	−2.538
	tf	lulogut44569	Forkhead/HNF-3-related transcription factor	3.00E-90	−2.960
	tr	lulogut21743	JAV05033.1 sodium/potassium-transporting atpase subunit beta-2-like protein	0	−2.991
	st	lulogutSigP-22,907	Acetylcholinesterase/Butyrylcholinesterase	4.00E-54	−5.397
	s/met/lipd	lulogutSigP-23,161	AAO22149.1 mammalian-like lipase	0	−5.688
	uk	lulogutSigP-18,032	Unknown product	NA	−5.917
**4d**	s/uk	lulogutSigP-14,897	hypothetical secreted protein precursor	1000	−3.861
	met/lipd	lulogut21836	JAV11771.1 lipid storage droplets surface-binding protein 1	0	−3.861
	s/met/nuc	lulogutSigP-26,492	JAV11299.1 deoxyribonuclease partial	0	−4.105
	s/protin	lulogutSigP-16,416	BPTI/Kunitz family of serine protease inhibitors	8.00E-17	−4.125
	met/carb	lulogut25316	Hexokinase	0	−4.299
	s/ uk	lulogutSigP-16,502	hypothetical conserved secreted protein precursor	NA	−4.926
	s/uk	lulogut36242	hypothetical secreted protein precursor	1000	−5.523
	s/	lulogutSigP-24,104	JAV08889.1 juvenile hormone binding protein in insects	0	−8.423
**12d**	detox	lulogut19743	JAV03807.1 metallothionein-2-like protein	2.00E-34	−2.909
	storage	lulogut21324	JAV06440.1 ovotransferrin partial	0	−3.778
	s/uk	lulogutSigP-16,502	hypothetical conserved secreted protein precursor	NA	−3.893
	s/protinh	lulogutSigP-16,416	BPTI/Kunitz family of serine protease inhibitors - signalP detected	8.00E-17	−3.902
	tm	lulogutSigP-15,657	nucleolar and coiled-body phosphoprotein 1 isoform X2 Drosophila ficusphila	4.00E-21	−4.086
	pm/protease	lulogut25198	JAV08757.1 trypsin	0	−4.383
	s/	lulogutSigP-24,035	JAV08413.1 secreted mucin	0	−4.536
	met/lipd	lulogut41307	JAV11511.1 ecdysteroid kinase	0	−6.148
**14d**	met/nuc	lulogut40330	Uridylate kinase/adenylate kinase	4E-59	−1.292

Legends: *Detox* oxidative metabolism/detoxification, *Nr* nuclear regulation, *Pm* protein modification, *S* secreted protein, *St* signal transduction, *Storage* storage protein, *Tf* transcription factor, *Tm* transcription machinery, *Tr* transporters and channels, *Uk* unknown protein. *Met/Carb* carbohydrate metabolism, *Met/Lipd* lipid metabolism, *Met/Nuc* nucleotide metabolism. *S/* other, *Protea* protease, *Protinh* protease inhibitor. *LFC* log_2_ Fold Change

### Functional profiles of the differentially expressed genes at different time points

**Fig. 4:**
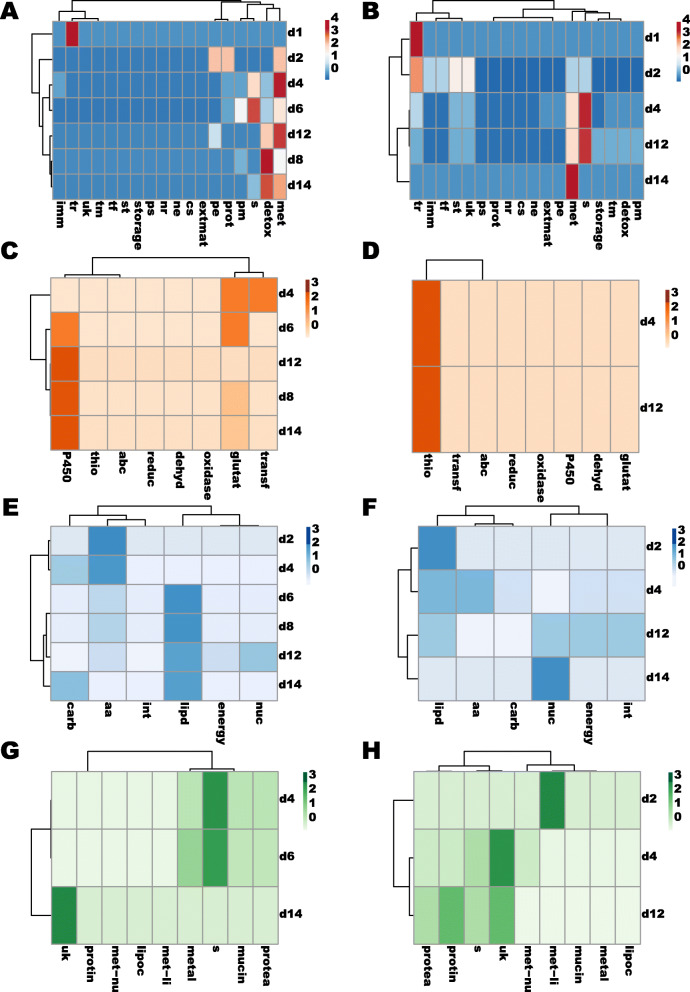
DE transcripts sorted by molecular functions. **a-b.** Heatmaps and cluster analyses depicting differences in the number of DE genes up-regulated (**a**) and down-regulated (**b**) by *Leishmania* infection belonging to different groups of molecular function. Legends: Cs: cytoskeleton; Detox: oxidative metabolism/detoxification; Extmat: extracellular matrix; Imm: immunity; Met: metabolism; Ne: nuclear export; Nr: nuclear regulation; Pe: protein export; Pm: protein modification; Prot: proteosome machinery; Ps: protein synthesis machinery; S: secreted protein; St: signal transduction; Storage: storage protein; Te: transposable element; Tf: transcription factor; Tm: transcription machinery; Tr: transporters and channels; Uk: unknown protein. **c-d.** Heatmaps and cluster analyses depicting differences in the number of DE genes up-regulated (**c**) and down-regulated (**d**) by *Leishmania* infection, belonging to different sorts of oxidative metabolism/detoxification molecular function. Legends: Dehyd: dehydrogenase; Glutat: glutathione s-transferase; P450: cytochrome P450; Oxidase: oxidase/peroxidase; Reduc: reductase; Abc: Transporter ABC superfamily; Thio: thioredoxin binding protein; Transf: sulfotransferase. **e-f.** Heatmaps and cluster analyses depicting differences in the number of DE genes up-regulated (**e**) and down-regulated (**f**) by *Leishmania* infection belonging to different sorts of metabolism molecular function, respectively. Legends: Aa: amino acid metabolism; Carb: carbohydrate metabolism; Energy: energy production; Int: intermediate metabolism; Lipd: lipid metabolism; Nuc: nucleotide metabolism. **g-h.** Heatmaps and cluster analyses depicting differences in the number of DE genes up-regulated (**g**) and down-regulated (**h**) belonging to different sorts of secreted protein molecular function. The heatmaps are color-coded according to the legends on the right. Legends: S: other; Metal: metalloprotease; Lipoc: lipocalin; Met-li: lipase; Met-nu: nuclease; Mucin; Protea: protease; Protin: protease inhibitor; Uk: unknown protein. DE was considered significant for transcripts displaying LFC either lower than − 1 or higher than 1 and FDR q-value lower than 0.05

Although the midgut genes up- and down-regulated by *Leishmania* infection exhibited different expression patterns across time points (Additional file 10: Figure S3), such DE genes belonged to the same functional groups for the most part (Fig. 4 and Tables 3 and 4 and Additional file 11: Table S8). Regarding the up-regulated genes, 28, 38, and 18% belonged to the detoxification (detox), metabolism (met), and secreted (s) protein molecular functions, respectively (Fig. 4a and Table 3). In fact, the enrichment of such molecular functions amongst the up-regulated genes was consistent through time (Fig. 4a and Table 3): between 2d through 14d for the metabolism function; and between 8d and 14d for the detoxification function. For the secreted protein category, the enrichment of up-regulated genes was more restricted to 4d and 6d (Fig. 4a and Table 3). At earlier time points (1d and 2d), the few up-regulated genes perform different functions ranging from transporter channels (tr, 1d) to proteosome machinery (prot, 2d; Fig. 4a and Table 3). Regarding midgut genes downregulated by the *Leishmania* infection, 34% of these genes belonged to the metabolism (22%) and secreted protein (12%) functional groups (Fig. 4b and Table 4). Both categories were consistently enriched on 4d, 12d, and 14d (Fig. 4b and Table 4). At earlier time points (1d and 2d), transporter channels (tr, 1d and 2d) and signaling transduction (st, 2d) were the most enriched molecular functions amongst the down-regulated genes (Fig. 4b and Table 4). All the molecular functions identified over all time points were matched by analogous GO terms (Additional file 12: Table S9 and Additional file 13: Table S10).

In order to investigate in-depth the functional profiles of the DE genes, we broke down the most predominant functional classes into subclasses. For the midgut DE genes belonging to the detoxification molecular function (detox), the cytochrome P450 gene family encompassed 76% of the up-regulated genes (Fig. 4c and Table 3 and Additional file 11: Table S8). Such genes were consistently upregulated between 6d and 14d (Fig. 4c and Table 3). In contrast, the down-regulated genes belonging to the detoxification molecular function were enriched in metallothioneins (4d and 12d, thio; Fig. 4d and Table 4). As far as the DE midgut genes belonging to the metabolism function, 55% of the up-regulated genes were related to the metabolism of lipids (lipd; Fig. 4e and Table 3) which was consistently the most predominant between 6d and 14d (Fig. 4e). Among the down-regulated genes performing metabolic functions (Fig. 4f and Table 4), 31% participated in the metabolism of lipids (lipd) at early time points (2d and 4d) or nucleotides (nuc) at later time point (12d and 14d, Fig. 4f and Table 4). Regarding the DE midgut genes encompassing the secreted proteins (Fig. 4g-h), 50% of those up-regulated belonged to the ‘other category’ (s, multiple protein functions) that was enriched in transcripts of insect allergen proteins (Fig. 4g; Table 3 and Additional file 11: Table S8), also known as microvilli proteins. Although the insect allergens, along with the mucins, and to a lesser extent metalloproteases (metal), were more predominant on 4d and 6d (Fig. 4g and Table 2), up-regulated transcripts encoding proteins of unknown function were enriched at 14d, a later time point (Fig. 4g and Table 3). Among the down-regulated transcripts encoding secreted proteins, 44% belonged to the unknown function (31%, uk) and “other” (17%, s) categories (Fig. 4h and Table 4). The “other” category (s) was consistently downregulated on 4d and 12d (Fig. 4h and Table 4) and was enriched in transcripts encoding juvenile hormone (JH) binding proteins as well as attacin (Table 4). Transcripts of secreted proteins related to the digestion of lipids (met-li) were down-regulated on 2d (Fig. 4h and Table 4).

## Discussion

In this work, we carried out a broad RNA-Seq investigation to assess the effects of *Leishmania* infection in sand fly midgut gene expression. As a sand fly genome is not available for use as a reference for read mapping, all the reads obtained were assembled de novo into 13,841 putative transcripts. Additionally, we manually curated 740 transcripts potentially participating in biological processes important for midgut homeostasis, such as immune responses, digestion, and chitin metabolism. This significant number of likely unique transcripts brings to light multiple homologs to complement the gene set annotated in the cDNA transcriptome study of whole *P. papatasi* sand flies [20]. Altogether, these transcripts will be of great use for gene prediction and annotation of sand fly genome projects.

We also used the 13,841 transcripts as a reference for gene expression quantification and comparisons between infected and uninfected samples. Out of seven time points, only about 1% of the genes were differentially expressed (113 genes) by *Leishmania* infection, highlighting the extent of the adaptation of *Le. infantum* to its natural vector, the sand fly *Lu. longipalpis*.

Multiple midgut genes displaying differential expression upon *Leishmania* infection in cDNA libraries of *Le. infantum*-infected *Lu. longipalpis* midguts [18] were also differentially expressed in our RNA-Seq libraries. For instance, all four insect allergen proteins (microvilli proteins), multiple digestive enzymes (proteases and peptidases), an astacin-metalloprotease, as well as a peritrophic matrix protein were differentially regulated by *Leishmania* infection in both studies [18].

The limited influence of *Leishmania* in midgut gene expression as observed in this study was further investigated by PC analysis. As indicated by PC1, most of the variance (77%) in the transcriptional levels across midgut samples was caused by the presence (or lack of) blood in the midgut, sorting out the early (d1 and d2; blood engorged) from late (d4 onwards; blood passed) time points. Even though PC2 (6.4%) and PC3 (4.1%) exhibited similar levels of variance, PC3 accounted for most of the variance sorting infected from uninfected midguts, and likely represents the differential expression of the 113 genes modulated by *Leishmania* infection. These findings also suggest that other factors not controlled for by the experimental design accounted for the variance observed in PC2. Along these lines, it is noteworthy that *Leishmania* infection in the sand fly midguts also modify the microbiota composition [22], which may also have affected gene expression in the midgut samples.

It is worth noting that multiple genes DE upon *Leishmania* infection were unique to a particular time point, being more pronounced in early time points. This phenomenon may be explained by the enrichment of different *Leishmania* stages at specific time points. For instance, time points 1d, 2d, 4d, and 6d were enriched in amastigotes and transitional stages, and procyclic, nectomonad, and leptomonad promastigotes, respectively. From 6d onwards, *Leishmania* parasites undergo metacyclogenesis. Hence, there is a gradual increase in the proportions of metacyclic compared to leptomonad promastigotes through time, which can explain the overlap of DE genes between midguts on 8d and the other late time points. Surprisingly, we observed a burst of down-regulated DE genes on 12d that was not observed on 14d. At both time points the midgut infection is very similar as far as parasite stage and density, a phenomenon that needs to be further investigated.

In order to complete its life cycle in the sand fly midgut, *Leishmania* needs not only to develop and differentiate into the infective metacyclic stage, but also to escape the barriers imposed by the sand fly midgut early in the infection (day 1–5). During this period, *Leishmania* needs to shield itself against the harmful actions of the proteolytic enzymes [9], avoid the immune system [10, 11], escape from the peritrophic matrix [12, 13], and attach to the midgut epithelium [14]. At these early time points, most of the sand fly DE genes were downregulated by large fold changes. On day 4, multiple sand fly genes encoding digestive enzyme as well as a peritrophic matrix protein were downregulated, pointing to parasite manipulation of the barriers imposed by the sand fly midgut in order to survive. Such down-regulation might also lead to increased availability of nutrients for the parasites. Along the same lines, it is important to highlight that the presence of *Leishmania* in the sand fly midgut leads to the down regulation of genes potentially involved with the control of gene expression. For instance, the transcription factor Forkhead/HNF-3, which is involved with midgut regeneration [23], and nutrient transport and absorption [24], were down-regulated on day 2. Accordingly, we have also observed down-regulation of sand fly amino acid and trehalose transporters on 4d after *Leishmania* infection. Transcripts for metallothionein-2-like protein were also down-regulated at the same time point. The expression levels of these proteins are used as a proxy of heavy metals absorption [25]. Hence, their down-regulation in *Leishmania*-infected midguts suggests that these parasites reduce nutrient uptake by the sand fly midgut epithelium. Along the same lines, genes encoding proteins associated with metabolism of hormones, such as the juvenile hormone and ecdysone, were down-regulated on days 4 and 6. Such hormone levels change during blood digestion [26], and relevantly control the expression of midgut serine proteases [27–29], which are also down-regulated upon *Leishmania* infection on days 4 and 6. Together, these data suggest that the sand fly transcription factor Forkhead/HNF-3 as well as hormone metabolic enzymes might be key targets to control *Leishmania* infection early on.

As the remains of the digested blood is flushed out and the parasites detach from the epithelium [14], the parasites undergo metacyclogenesis from day 6 onwards, migrating to the anterior midgut and differentiating into infective forms [8]. At this late period in the infection, midgut barriers to *Leishmania* development are unknown or negligible. The parasites seem to multiply freely, secreting a massive amount of carbohydrates (fPPG) that blocks blood intake and allows the parasites to be regurgitated into the skin [30, 31]. Most of the sand fly DE genes late in infection (day 8 onwards) were upregulated by small fold change differences in response to *Leishmania*. Most of these genes encode proteins that participate in detoxification of xenobiotics (cytochrome P450) and metabolism of lipids. At these time points, it seems plausible that the massive amount of parasites, reaching 120,000 cells on average on day 14 [21], might be indirectly modulating sand fly gene expression by the release of cell membranes and metabolites from dead parasites and *Leishmania*-derived exosomes [32] throughout metacyclogenesis. Interestingly, the presence of *Leishmania* is undetected by the midgut immune system of the sand fly during this period. This is also noted at early time points with the exception of day 4 where the down-regulation of a gene encoding attacin, an antimicrobial peptide [33], was observed. The lack of *Leishmania* detection by the immune system may constitute another adaptation of *Le. infantum* to survive in *Lu. longipalpis* midguts.

## Conclusion

Overall, the presence of *Le. infantum* in the midgut of its natural vector has direct and indirect effects on sand fly midgut gene expression. On one hand, these parasites appear to manipulate gene expression early on to weaken developmental barriers imposed by the midgut. On the other hand, *Leishmania* behaves like a commensal later in the infection, and changes in the sand fly gene expression caused by the parasites seem to be an indirect consequence of the massive amount of the parasites inside the anterior portion of the midgut. Altogether, our findings expose a fine-tuned relationship that has evolved over time to ensure optimal survival and transmission of the *Leishmania* parasite by vector sand flies.

## Methods

### *Leishmania* parasites, parasite load assessment, sand fly blood feeding and infection, and midgut dissection and storage

*Lutzomyia longipalpis* sand flies were obtained from a sand fly colony maintained at the insectary facility, Laboratory of Malaria and Vector Research, National Institute of Allergy and Infectious diseases (LMVR/NIAID). Sand fly infection and *Leishmania* counts were performed as described in our companion manuscript [21]. As controls, *Lu. longipalpis* also fed on uninfected heparinized dog blood at the same time. Blood of beagle dogs was provided by the Division of Veterinary Resources (DVR/NIH) under animal use protocol ORS 17 and withdrawn on the same day of the experiments. After feeding, fully fed females were sorted and given 30% sucrose solution ad libitum. In order to assess how gene expression in sand fly midguts is affected by *Leishmania* growth and differentiation, *Le. infantum* infected-*Lutzomyia longipalpis* midguts were dissected at days one, two, four, six, eight, twelve, and fourteen after blood feeding on RNAse Free PBS (1X) for RNA-Seq library construction in triplicate and compared to midguts fed on uninfected blood at the same time points.

### RNA extraction and quality control

For total RNA extraction, the PureLink RNA Mini Kit (Life Technologies, Carlsbad) was used as described in [21], following the manufacturer’s recommendations. RNA amounts and purity were assessed using a Nanodrop spectrophotometer (Nano Drop Technologies Inc., Wilmingtom; ND-1000), and quality control was further evaluated using a Bioanalyzer (Agilent Technologies Inc., Santa Clara, CA; 2100 Bioanalyzer), using the Agilent RNA 6000 Nano kit (Agilent Technologies) and following the manufacturer’s recommendations. Out of the 48 samples, only 1 displayed a low RIN value (RNA integrity number = 6.7; 14d Pi, replicate 3).

### RNA-Seq library preparation and deep sequencing

The RNA-Seq libraries were prepared using the NEBNext® Ultra™ RNA Library Prep Kit for Illumina (New England Biolabs, Ipswick MA), following manufacture’s recommendation, for Single Ended sequencing by HiSeq 2500 (Illumina, San Diego, CA) of 125 nucleotides reads (SE - 125), and sequenced at the NC State University Genomic Science Laboratory. All the libraries gave rise to high quality data and robust expression levels, except one replicate of the 2d PBM and another of the 12d PBM time points, which were excluded from further analyses.

### Bioinformatic pipeline and de novo assembly

RNA-seq data trimming and mapping were described elsewhere [21]. De novo assembly from high quality reads were a result of both Abyss (kmers from 21 to 91 in 10-fold increments) and Trinity (V2.1.1) assemblers. The output reads were assembled using an iterative blast and CAP3 pipeline as previously described [34]. Sequences sharing more than 95% identity at nucleotide level were assumed to be alleles of the same genes and clustered together as unique transcripts. Protein coding sequences were defined based on prediction of open reading frame signatures, identification of signal peptide-coding sequence, and by similarities searches in the Refseq invertebrate database from the National Center for Biotechnology Information (NCBI), sequences from Dipterans deposited at NCBI’s Genbank and from SwissProt. Protein-coding sequences were annotated upon similarity matches to various databases, including Swissprot, Gene Ontology, KOG, Pfam, Drosophila mRNA transcripts, Virus, and SMART, Refseq-invertebrates and the Diptera subset of the GenBank sequences obtained by querying diptera [organism] and retrieving all protein sequences. Raw reads were deposited on the Sequence Read Archive (SRA) of the National Center for Biotechnology Information (NCBI). This Transcriptome Shotgun Assembly project has been deposited at DDBJ/ENA/GenBank under the accession GITU00000000. All sequences used in this work and their corresponding accession numbers are in Additional file 15: Table S12.

Reads were mapped to the generated dataset using the RNA-Seq by Expectation Maximization (RSEM) version 1.3.0, Bowtie version 2–2.2.5, and samtools version 1.2 [35]. Differential expression was analyzed using the Bioconductor package DeSeq2 vs 3.8 [36], using default parameters and the shrinks log2 fold-change (FC) estimation [36, 37]. Genes exhibiting sum of counts inferior to 10 across all time points were removed. Differential expression was considered statistically significant under adjusted *p*-value (P-adj) lower than 5% (*p* < 0.05) and log_2_ fold change higher or lower than 0.5. The whole dataset can be found in Additional file 14: Table S11.

### Filtering and annotation of specific gene families

Transcripts involved in biological processes related to immunity, digestion, and chitin metabolism were filtered upon tBLASTn searches against *Drosophila melanogaster* [38] and sand fly [18, 19] immune-related genes, sand flies digestive enzymes [18, 19], and *Tribolium castaneum* [39–42] and sand fly [12, 13] chitin metabolism homologs. Other members of such gene families were further filtered by manual screening of similar KOG and Swiss databases matches. Only transcripts exhibiting e-values lower than 10^− 5^ against most of the databases were annotated. For the annotation of mucin sequences, we relied on motif identification by the automated pipeline described above.

### Data and statistical analyses

Principal component analysis (PCA) were carried out with the PAST3 software [43], based upon log_2_ TPMs. Statistical analyses were carried out with Prism 7 (GraphPad Software Inc). Venn diagram results were obtained with Venny 2.1 (http://bioinfogp.cnb.csic.es/tools/venny/), and gene function heatmaps were obtained using the ClustVis tool ([44]; https://biit.cs.ut.ee/clustvis/). Transcript count heatmap, bubble plots, and volcano plots were obtained with the gplots and ggplot2 packages and constructed with the R software.

### nCounter XT gene expression assessment

Gene expression validation was carried out using the nCounter probe-based hybridization assay (NanoString Technologies Inc., Seattle, WA), following the manufacturer’s recommendation. Forty-two sand fly genes were randomly chosen (Additional file 6: Table S3) for probe design and hybridized against 100 ng of each RNA sample, resulting in three biological replications per time point. Raw output data were analyzed using the nSolver software (NanoString Technologies), normalizing the results against the counts for all 42 genes. Only genes detected by the nCounter were considered for comparisons to RNA-Seq data. For a gene to be considered nCounter-detected [45], the average counts for the experimental gene had to be significantly higher than the average counts of eight negative control by Mann Whitney U test (p < 0.05) in at least one of the treatments (infected or uninfected). The expression of the detected genes in each time point was used for expression comparisons with the RNA-Seq expression results for the correspondent genes. For these comparisons, only genes displaying average TPM of at least 1 in one of the treatments were considered. Fold change correlations were determined by plotting the log_2_ ratio of the infected over the uninfected expression values for RNA-Seq (TPMs) and nCounter (normalized counts) and calculating the linear regression coefficient.

## Supplementary information

**Additional file 1 **: **Table S1** Summary of the *Lu. longipalpis* midgut transcripts.

**Additional file 2 **: **Figure S1** Heatmap displaying the expression profiles and cluster analyses of the midgut transcripts across seven time points in uninfected and *Leishmania*-infected samples. The 10,000 most highly expressed transcripts are depicted.

**Additional file 3 **: **Table S2** Summary of the overall percentage of contigs (% of contigs) or abundance (%TPM) for all time points. The distribution of the mapped reads to the functional classification are highlighted.

**Additional file 4 **: **Table S3** Annotation of transcripts participating in biological processes related to immunity, digestion, and peritrophic matrix/chitin metabolism.

**Additional file 5 **: **Table S4** Principal component analysis output for comparisons between average transcriptional expression amongst time points as well as for individual replicates.

**Additional file 6 **: **Figure S2** Principal component analysis (PCA) describing the position of each replicate for each midgut time point in the expression space. (**A**) Expression space was generated based on the log2 of TPMs using the 10,000 most expressed transcripts across libraries. The Eigenvalues and % variance for PC1 and PC3 were 5632.97 and 60% and 321.15 and 3.4%, respectively. (**B**) Expression space between PC1 and PC2. The Eigenvalues and % variance for PC2 were 670.05 and 7.1%, respectively. The color codes labeling each time point were as follow: Aqua (1d); Royal Blue (2d); Sea Green (4d); Sandy Brown (6d); Saddle Brown (8d); Red (12d); and Fuchsia (14d). The triangle and circle shapes represent *Leishmania*-infected and uninfected samples, respectively.

**Additional file 7 **: **Table S5** nCounter probes, counts, and expression comparisons with RNA-Seq TPMs.

**Additional file 8 **: **Table S6** Gene sets displaying differential gene expression at each time point.

**Additional file 9 **: **Table S7** Genes uniquely differentially expressed at each time point.

**Additional file 10 **: **Figure S3** Volcano plots depicting the differentially expressed (DE) transcripts at each time point. (**A-G**). DE transcripts at 1d, 2d, 4d, 6d, 8d, 12d, and 14d, respectively. Only transcripts exhibiting q-values lower than 0.05 are shown. Transcripts displaying fold change greater or lower than 2 (− 1 < LFC > 1) are color coded, as follow: Aqua (1d); Royal Blue (2d); Sea Green (4d); Sandy Brown (6d); Saddle Brown (8d); Red (12d); and Fuchsia (14d). LFC scale is color coded in gray (top right). In black, transcripts not significant at − 1 < LFC > 1.

**Additional file 11 **: **Table S8** Functional analyses of differentially expressed genes.

**Additional file 12 **: **Table S9** Gene Ontology (GO) enrichment for the up-regulated genes at each time point.

**Additional file 13 **: **Table S10** Gene Ontology (GO) enrichment for the down-regulated genes at each time point.

**Additional file 14 **: **Table S11** Transcriptional and bioinformatics description of the *Lu. longipalpis* midgut transcripts.

**Additional file 15 **: **Table S12**. Accession numbers corresponding to all transcripts (sequences) reported in this work.

## Data Availability

The datasets generated and/or analyzed during the current study are available at DDBJ/ENA/GenBank under the accession GITU00000000. All sequences used in this work and their corresponding accession numbers are in Additional file 15: Table S12.

## Abbreviations

Forkhead/HNF-3: Hepatocyte nuclear factor 3/fork head
TPM: Transcripts per million
PBM: Post blood meal
Pi: Post infection
fPPG: Filamentous proteophosphoglycan
DE: Differentially expressed
PCA: Principal component analysis
LFC: Log2 fold change
ORF: Open reading frame
GO: Gene ontology
SRA: Sequence Read Archive
NCBI: National Center for Biotechnology Information
PBS: Phosphate buffer saline
ROX: Reactive oxygen species
PM: Peritrophic matrix
CPAP: Cuticular proteins analogous to peritrophins
