# Supplementary figures and images for: Leishmania infection induces a limited differential gene expression in the sand fly midgut
Source: BMC Genomics. 2020 Sep 4;21:608. doi: 10.1186/s12864-020-07025-8 (PMC7487717; doi:10.1186/s12864-020-07025-8)

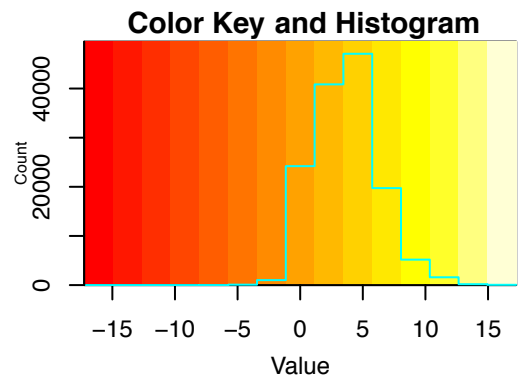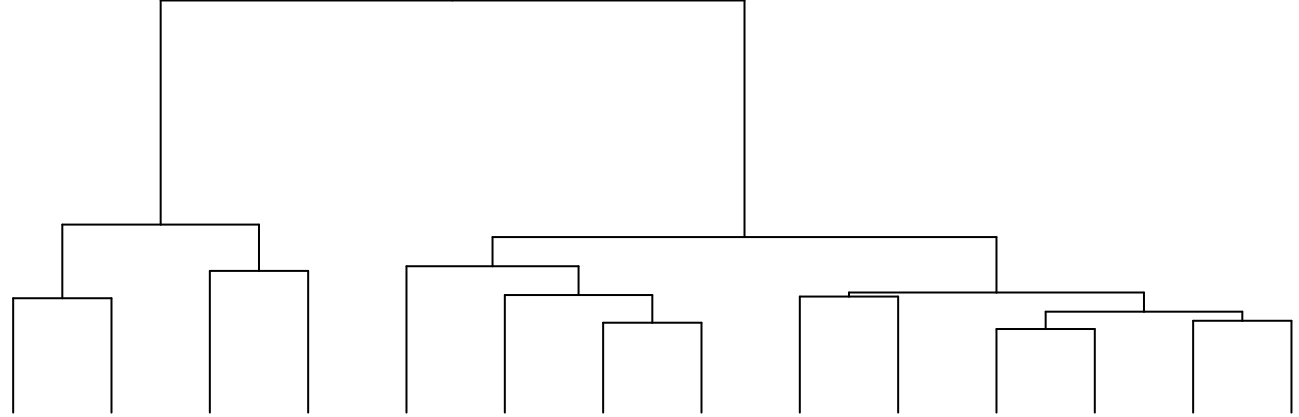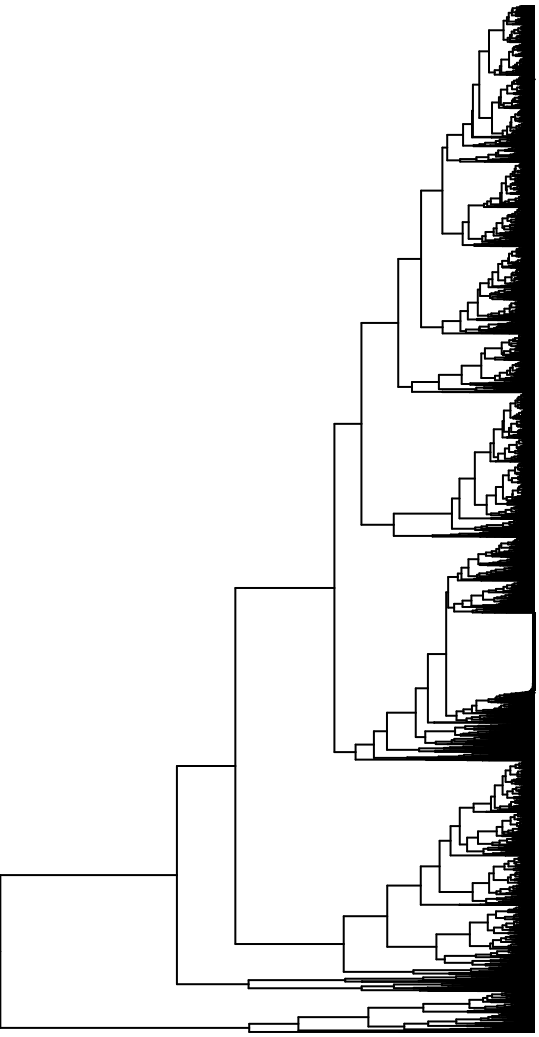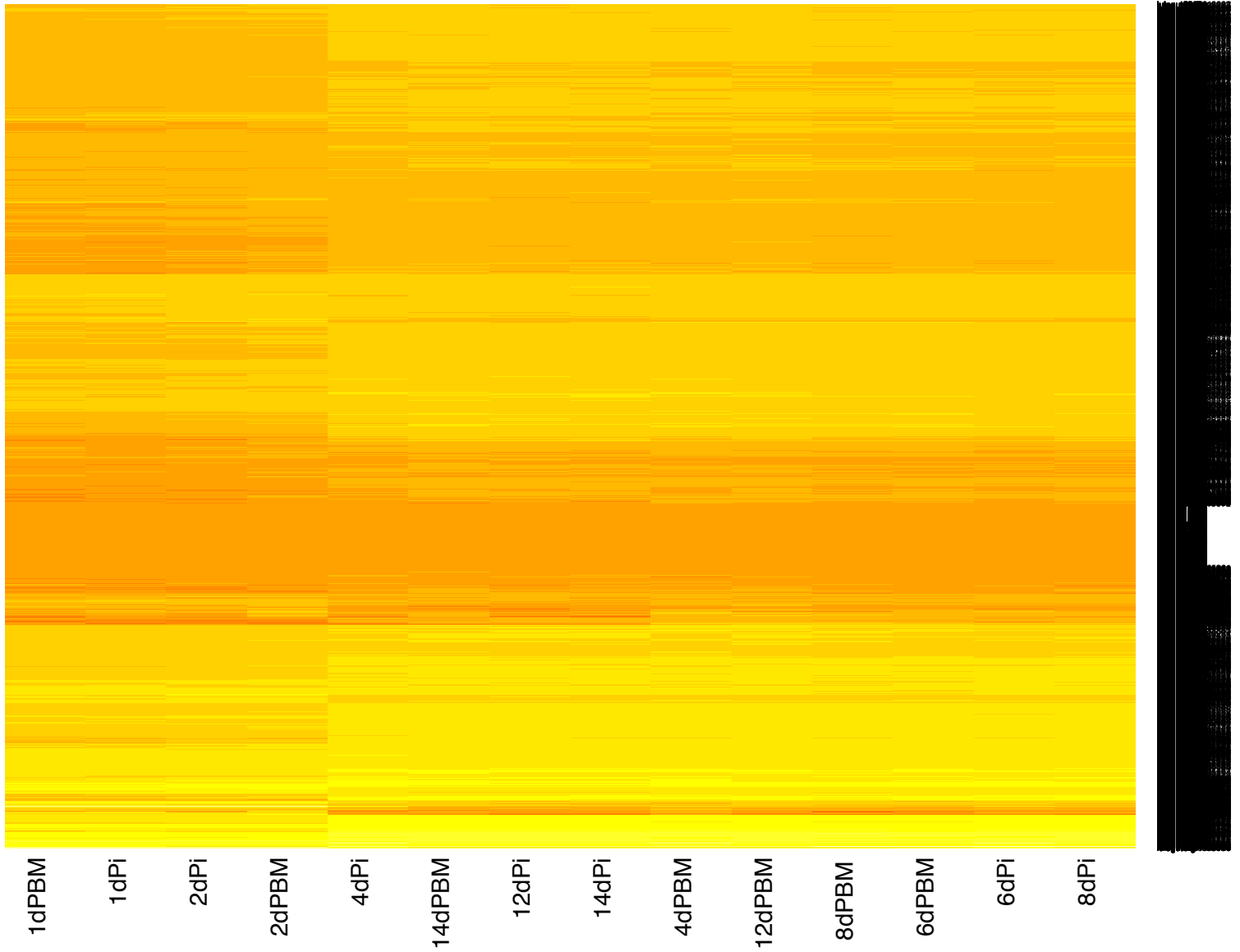

Supplement: Supplementary file 2 — Additional file 2 : Figure S1 Heatmap displaying the expression profiles and cluster analyses of the midgut transcripts across seven time points in uninfected and Leishmania-infected samples. The 10,000 most highly expressed transcripts are depicted. [file 12864_2020_7025_MOESM2_ESM.pdf]

**A**

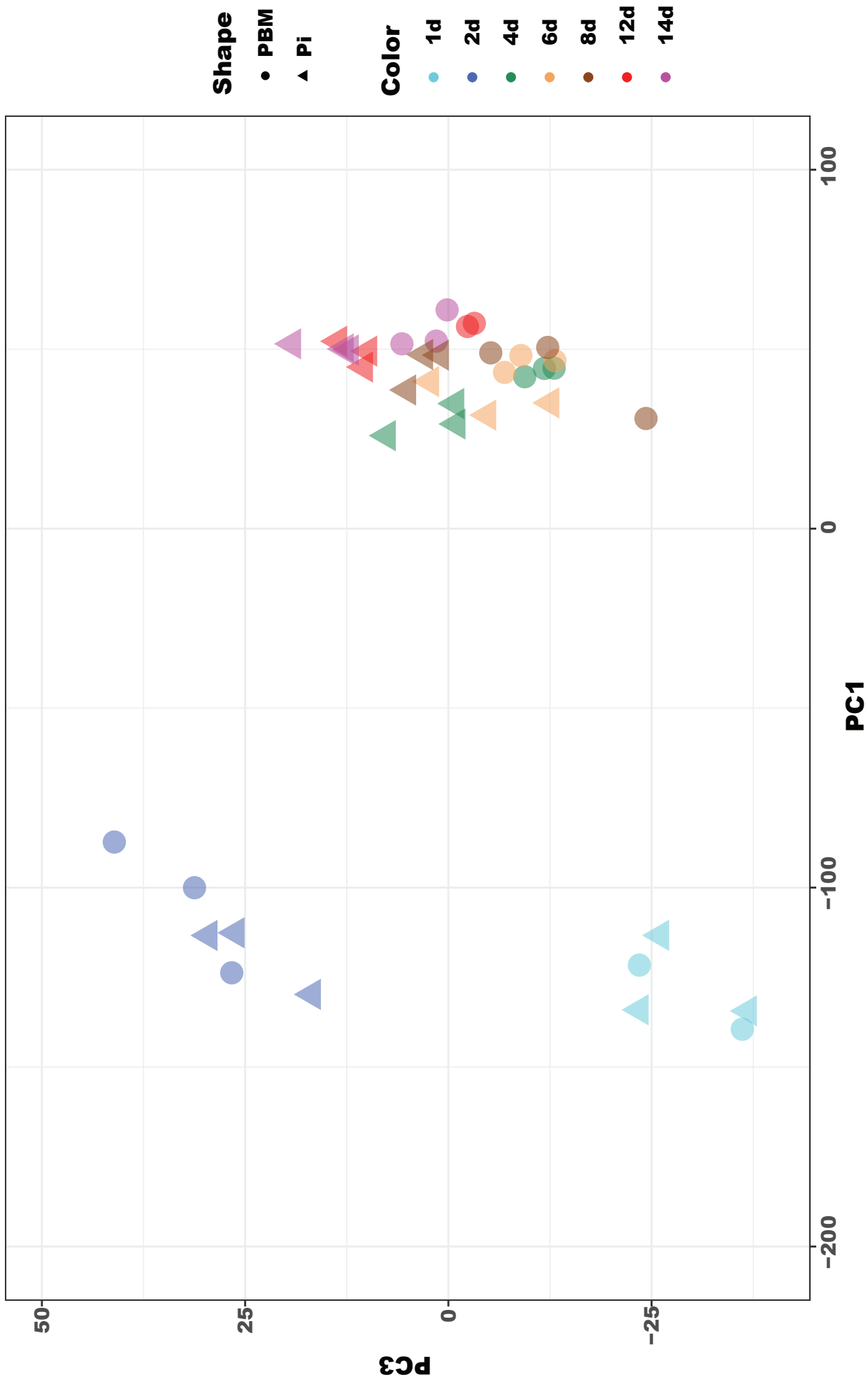

**B**

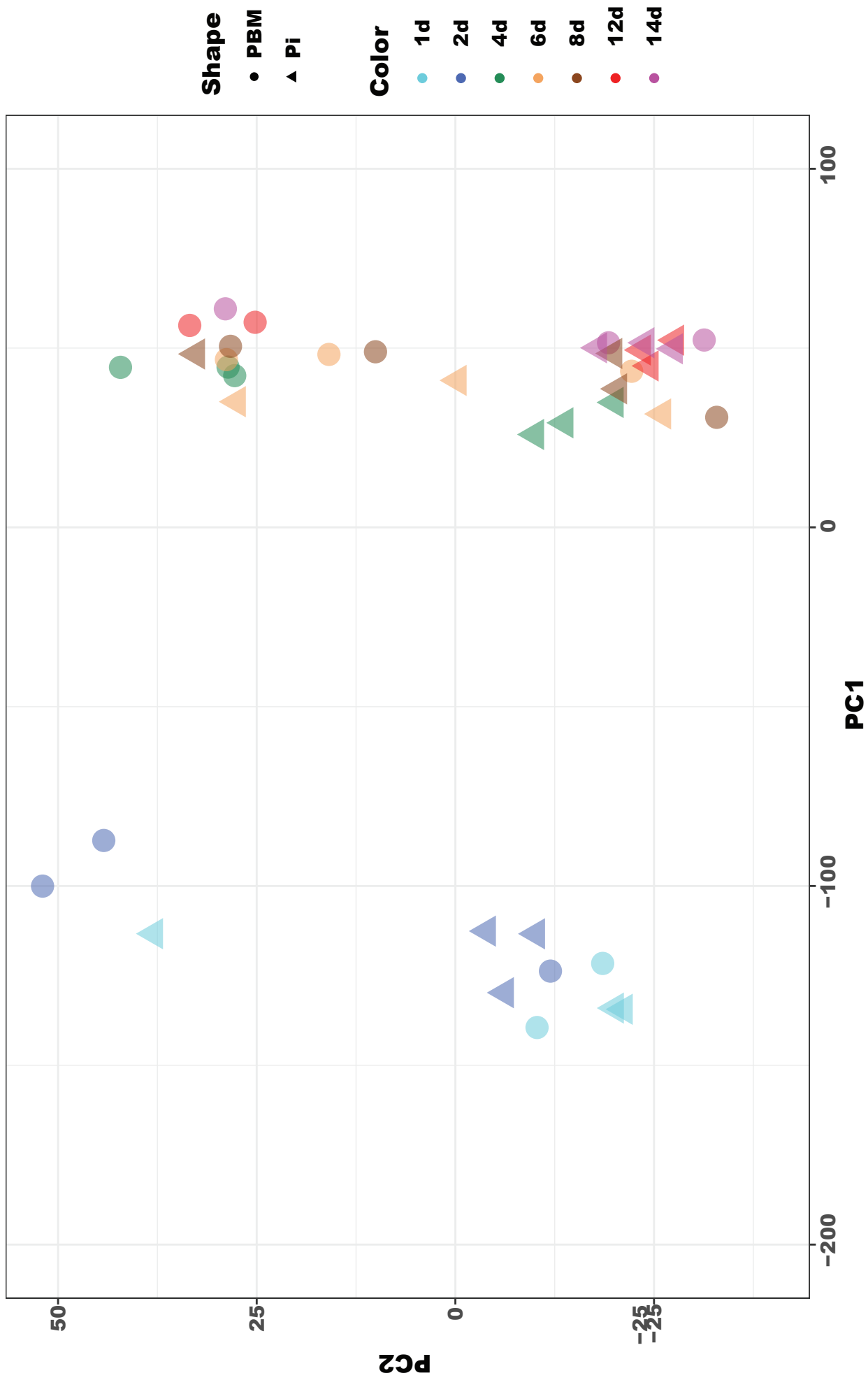

Supplement: Supplementary file 6 — Additional file 6 : Figure S2 Principal component analysis (PCA) describing the position of each replicate for each midgut time point in the expression space. (A) Expression space was generated based on the log2 of TPMs using the 10,000 most expressed transcripts across libraries. The Eigenvalues and % variance for PC1 and PC3 were 5632.97 and 60% and 321.15 and 3.4%, respectively. (B) Expression space between PC1 and PC2. The Eigenvalues and % variance for PC2 were 670.05 and 7.1%, respectively. The color codes labeling each time point were as follow: Aqua (1d); Royal Blue (2d); Sea Green (4d); Sandy Brown (6d); Saddle Brown (8d); Red (12d); and Fuchsia (14d). The triangle and circle shapes represent Leishmania-infected and uninfected samples, respectively. [file 12864_2020_7025_MOESM6_ESM.pdf]

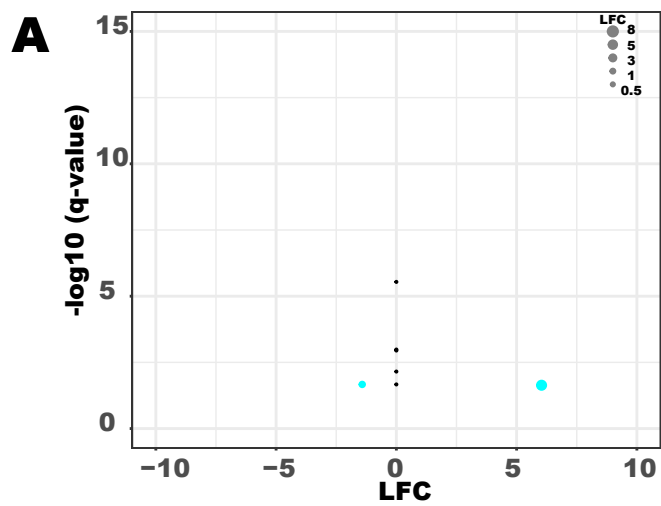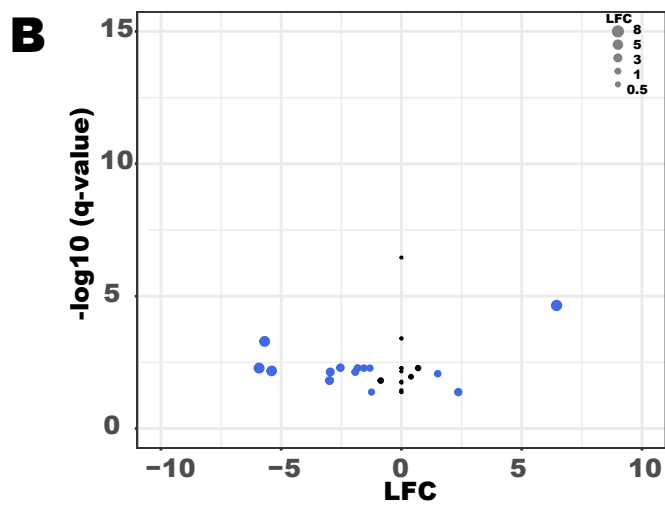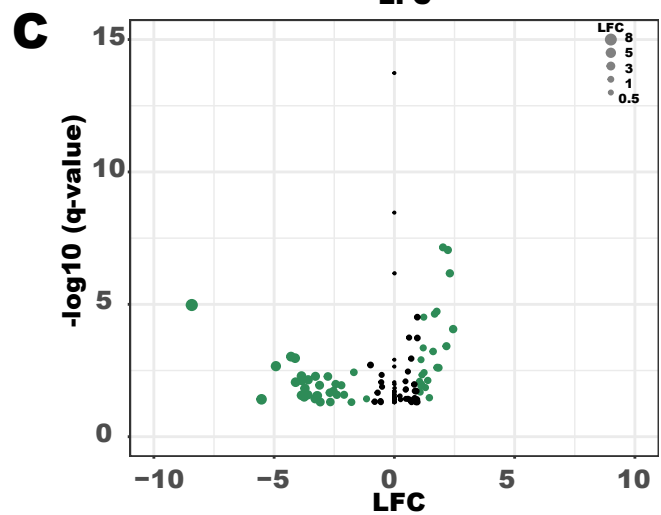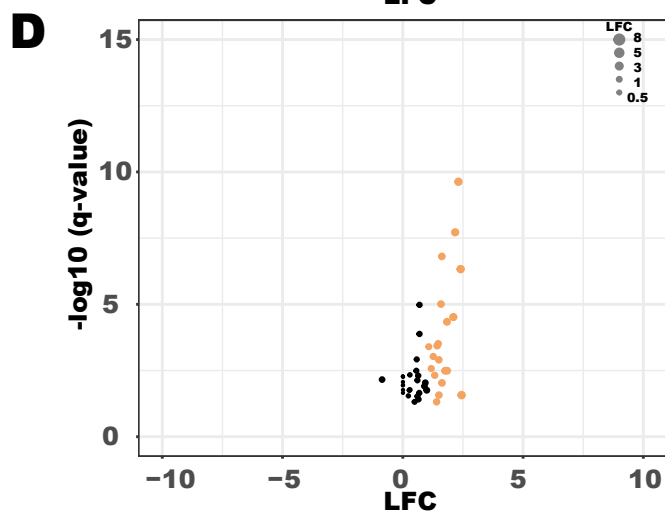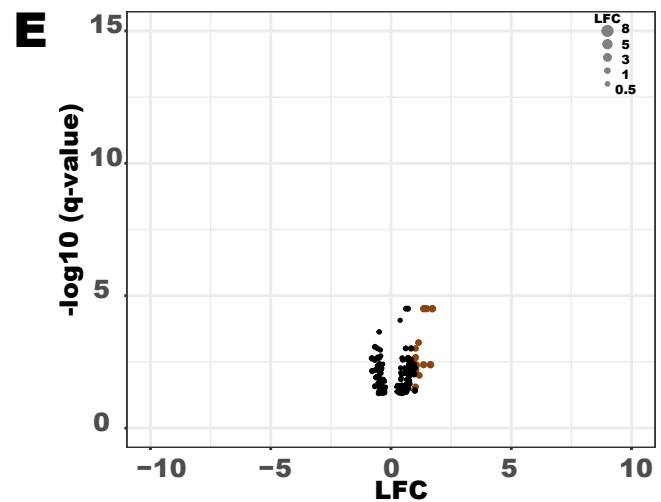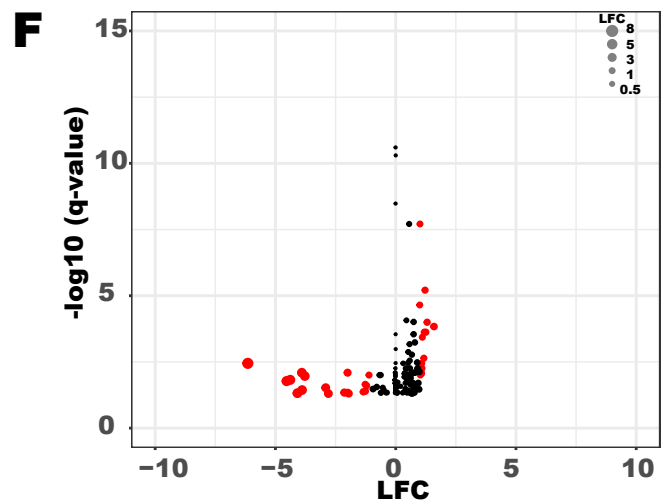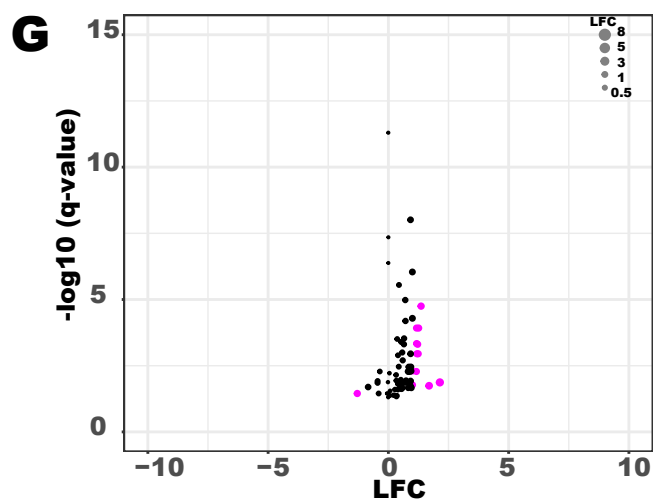

Supplement: Supplementary file 10 — Additional file 10 : Figure S3 Volcano plots depicting the differentially expressed (DE) transcripts at each time point. (A-G). DE transcripts at 1d, 2d, 4d, 6d, 8d, 12d, and 14d, respectively. Only transcripts exhibiting q-values lower than 0.05 are shown. Transcripts displaying fold change greater or lower than 2 (− 1 < LFC > 1) are color coded, as follow: Aqua (1d); Royal Blue (2d); Sea Green (4d); Sandy Brown (6d); Saddle Brown (8d); Red (12d); and Fuchsia (14d). LFC scale is color coded in gray (top right). In black, transcripts not significant at − 1 < LFC > 1. [file 12864_2020_7025_MOESM10_ESM.pdf]
